# Self-reactive T cells induce and perpetuate chronic relapsing arthritis

**DOI:** 10.1186/s13075-020-2104-7

**Published:** 2020-04-28

**Authors:** Jonatan Tuncel, Jens Holmberg, Sabrina Haag, Malin Hultqvist Hopkins, Lena Wester-Rosenlöf, Stefan Carlsen, Peter Olofsson, Rikard Holmdahl

**Affiliations:** 1 Division of Medical Inflammation Research, Department of Medical Biochemistry and Biophysics, Karolinska Institutet, Stockholm, Sweden; 2 Section for Medical Inflammation Research, BMCI11, Lund University, Lund, Sweden

**Keywords:** Chronic arthritis, Adoptive T cell transfer, PIA, Pristane, MHC class II, T cell depletion, RA

## Abstract

**Background:**

CD4^+^ T cells play a central role during the early stages of rheumatoid arthritis (RA), but to which extent they are required for the perpetuation of the disease is still not fully understood. The aim of the current study was to obtain conclusive evidence that T cells drive chronic relapsing arthritis.

**Methods:**

We used the rat pristane-induced arthritis model, which accurately portrays the chronic relapsing-remitting disease course of RA, to examine the contribution of T cells to chronic arthritis.

**Results:**

Rats subjected to whole-body irradiation and injected with CD4^+^ T cells from lymph nodes of pristane-injected donors developed chronic arthritis that lasted for more than 4 months, whereas T cells from the spleen only induced acute disease. Thymectomy in combination with irradiation enhanced the severity of arthritis, suggesting that sustained lymphopenia promotes T cell-driven chronic inflammation in this model. The ability of T cells to induce chronic arthritis correlated with their expression of Th17-associated transcripts, and while depletion of T cells in rats with chronic PIA led to transient, albeit significant, reduction in disease, neutralization of IL-17 resulted in almost complete and sustained remission.

**Conclusion:**

These findings show that, once activated, self-reactive T cells can sustain inflammatory responses for extended periods of time and suggest that such responses are promoted in the presence of IL-17.

## Introduction

Rheumatoid arthritis (RA) is a chronic relapsing autoimmune disease that affects approximately 0.5% of the population. Several lines of evidence indicate that CD4^+^ αβ T cells play a central role in the disease. First, certain alleles of MHCII, in particular, HLA-DRB1*04:01, *04:04, *04:05, and *01:01, are strongly associated with RA [1–3], as are genes encoding molecules controlling T cell activation and differentiation (*PTPN22, CD28 and CTLA4*) [4] [5]. Second, T cells with an activated phenotype have been found to be enriched in the synovial tissue of some RA patients and although shared clonotypes have been identified across different joints [6], it remains unclear whether such T cell clones recognize local antigens and, if so, whether they contribute to the pathogenesis in RA. Third, therapies that intercept with CD28:CD80/86 co-stimulation, such as CTLA-4-Ig (abatacept), have been proven successful for the treatment of RA. However, CD80/86 is broadly expressed on many cell types, including osteoclasts [7], which are important for bone resorption, and although it is likely that blocking of CD80/86 suppresses T cell activation, the mode of action of these drugs are still incompletely understood.

Hence, while there is a broad consensus that T cells are important in RA, it is still unclear what role they play during the various stages of the disease [8]. Self- or cross-reactive T cells may contribute at an early stage of RA by providing co-stimulation to B cells to promote the production of autoantibodies. To provide B cell help appears to be the primary responsibility of T cells in many animal models of RA, such as collagen-induced arthritis (CIA) [9–12] and K/BxN mice [13], in which transgenic T cells recognize the ubiquitously expressed self-antigen glucose-6-phosphate isomerase [14]. In both of these models, T cells are required for the activation of B cells to secrete pathogenic antibodies but are dispensable for the perpetuation of disease. In addition, aside from a few models of CIA induced by autologous collagen type II [15, 16], most antigen-induced models lack a chronic relapsing disease course making it impossible to address the impact of T cells for chronicity in these models.

In contrast, in adjuvant arthritis models, where the B cell response appears to be less critical, both the induction and perpetuation of arthritis are T cell-dependent. In classical adjuvant arthritis, for example, depletion of CD4^+^ T cells significantly ameliorates disease even after overt arthritis has been established. However, most of the adjuvant models are self-remitting [17], suggesting that prolonged T cell activation eventually leads to effector cell exhaustion and/or apoptosis. An exception is pristane-induced arthritis (PIA), which is a chronic model that, similar to RA, has a relapsing-remitting disease course [17]. Rats injected with pristane develop polyarticular arthritis with elevated levels of acute-phase reactants [17, 18] and IgG rheumatoid factors [19]. Since the inoculum in PIA is free from exogenous antigens, T cells are believed to recognize self-antigens in the context of MHCII [20, 21], and while self-antigens have been identified in PIA, e.g., collagen type XI [22] and hnRNP-A1 [23], the arthritogenic response appears to be polyclonal rather than directed towards a few major antigens [21–24].

PIA can be adoptively transferred using CD4^+^ T cells form pristane-injected rats [21]. However, whether T cells contribute to the perpetuation of chronic arthritis in PIA is still unclear since T cell-transferred rats recover from arthritis within a few weeks. In addition, while there is a strong genetic association between MHCII and the onset/severity of early PIA, there is no known association between MHCII and the development of chronic arthritis [20], suggesting that, besides the initial breach in tolerance, T cells might be dispensable for the perpetuation of chronic arthritis in this model.

Here, we revisited the question of whether T cells contribute to the perpetuation of chronicity in arthritis by examining the conditions required for optimal T cell transfer and by performing T cell ablation in rats with chronic disease.

## Materials and methods

### PIA induction and evaluation of arthritis

DA rats (originating from Zentralinstitut Fur Versuchstierzucht, Hannover, Germany, or, in separate experiments, Harlan Laboratories) were kept in a climate-controlled environment with 12-h light/dark cycles, housed in polystyrene cages containing wood shavings, and fed standard rodent chow and water ad libitum. Six to 10-week-old, sex- and age-matched female and male rats were used for all arthritis experiments. The induction and monitoring of arthritis have been described previously [17]. In brief, rats were injected intradermally (i.d.) at the base of the tail with 100 μl of pristane (2,6,10,14-tetramethylpentadecane) (Sigma-Aldrich). All disease assessments were performed blinded. Arthritis development was monitored in all four limbs using a previously described scoring system: 1 point (pt) was given for each swollen or red toe, 1 pt for each swollen midcourt, digit, or knuckle, and 5 pts for a swollen ankle (max 15 pts per paw; 60 pts per rat). All experiments were approved and performed in accordance with the guidelines from the Swedish National Board for Laboratory Animals and the European Community Council Directive (86/609/EEC).

### T cell adoptive transfer

Inguinal lymph nodes (iLNs) and spleen were harvested on day 14 after pristane injection. Tissues were passed through 40-μm cell strainers, cells were washed in PBS and then reactivated with Concanavalin A (3 μg/ml; Sigma-Aldrich) in complete DMEM medium, containing 5% FCS, HEPES (2.4 mg/ml), β-mercaptoethanol (3.9 μg/ml), and penicillin-streptomycin (10^4^ IU/ml penicillin, 10 mg/ml streptomycin; Invitrogen Life Technologies). After 72 h of incubation at 37 °C, cells were washed and resuspended in PBS. Each recipient was transferred with 2 × 10^7^ viable cells. Thymectomy was performed 3 weeks prior to transfer as previously described [25]. For whole-body irradiation, rats were exposed to a single dose of 6 Gy (600 rad) from a cesium-137 gamma-ray source, which depletes essentially all peripheral lymphocytes. Plasma concentration of cartilage oligomeric matrix protein (COMP) was determined by a competitive ELISA [26]. Levels of a1-acid glycoprotein (AGP) were measured with a soluble competitive radioimmunoassay (RIA) [27] using rat a_1_-acid glycoprotein.

### Isolation of CD4^+^ T cells for adoptive transfer

CD4^+^ T cells were isolated from iLNs of pristane-injected rats before ConA reactivation. LN cells were incubated with an excessive amount (50 μg/ml) of anti-CD8a (OX-8), anti-MQ (ED2), anti-CD45RA (OX-33), and anti-granulocytes (HIS48) antibodies and were then transferred to Petri dishes (5 × 10^6^ cells/plate), pre-coated with goat anti-mouse mAb (Jackson Immunoresearch 115-116-071; 10 mg/ml). After 45 min of incubation at 4 °C, suspended cells were counted and the purity of CD4^+^ T cells was assessed by flow cytometry (typically > 95% purity). CD4^+^ T cells were transferred at 2 × 10^7^ viable cells/recipient after ConA reactivation.

### Histological analysis

Paws from recipient rats were collected and decalcified with EDTA. Cryosections were stained with hematoxylin and were subjected to immunohistochemical staining with the following biotinylated antibodies: anti-CD4 (OX-35), anti-MQ (ED2), and anti-Granulocytes (HIS48) (all antibodies from Pharmingen), followed by streptavidin-peroxidase and visualized using the DAB substrate kit (DAKO, Denmark).

### Isolation of CD4^+^ T cells for mRNA expression analyses

Inguinal LN, mesenteric LN, and spleen cells were prepared from naïve and pristane-injected (day 13 after pristane administration) rats, resuspended at 10^7^ cells/ml in cold MACS buffer (0.5% BSA, 2 mM EDTA in PBS) and stained with mouse antibodies against NK1.1 (10/78), γ/δTCR (V65), CD45RA (OX33), and CD8a (OX8) for 10 min at 4 °C (all antibodies were purchased from Pharmingen). After wash, cells were resuspended in 1 ml of MACS buffer containing 50 μl of Dynabeads Pan Mouse IgG (DYLAN, Oslo, Norway), incubated for 45 min (rotating) at 4 °C and then washed again. NK cells, NK T cells, B cells, γ/δT cells, and CD8^+^ cells were removed by magnetic depletion. Non-depleted cells were washed once, resuspended in 80 μl MACS buffer, incubated for 15 min with 20 μl CD6-coated microbeads (Miltenyi Biotec, GTF, Goteborg, Sweden) and labeled cells were then positively selected on MS-columns (Miltenyi Biotec). Purity was assessed by flow cytometry (Fig. 3b).

### RNA extraction and expression analyses

CD4^+^ T cells were resuspended in 300 μl of RLT buffer (QIAGEN Nordic, Ballerup, Denmark), containing 10 μl/ml β-mercaptoethanol. Automated RNA isolation was performed on a QIACube robot using the RNeasy extraction kit (Qiagen) with on-column DNase I digestion (Qiagen). RNA samples were diluted to 10 ng/ml in DEPC-treated water (Ambion). Complementary DNA (cDNA) was synthesized using the High Capacity cDNA Reverse Transcription Kit (Applied Biosystems, Foster City, CA, USA). Primers (Additional file 1: Table S1) were designed in Primer-BLAST (ncbi.nlm.nih.gov/tools/primer-blast/index.cgi) or obtained from the RTPrimerDB (medgen.ugent.be/rtprimerdb). SYBR-Green PCR master mix (Applied Biosystems, Foster City, CA, USA) was used for all PCRs according to the manufacture’s recommendation. Expression analyses were performed on an ABI Prism 7900 HT (Applied Biosystems). Specificity and efficiency of primers were validated using the absolute quantification method. Expression of targets was normalized to the expression (geometric mean) of three reference genes (*Arbp*, *Act,* and *Gusb*).

### Antibodies and immunosuppressive reagents

Anti-mouse IL-17A antibody (clone 17F3; mouse IgG1) and isotype control antibody (clone MOPC-21) were obtained from Bio X Cell (West Lebanon, NH, USA). Cross-reactivity to rat IL-17 has been evaluated previously [24]. Anti-rat αβTCR antibodies (clone R73) were produced from hybridoma. All antibodies were administrated i.v. in 0.2 ml sterile PBS (1 mg/ml) at various time-points after pristane injection as indicated in the respective figure. Etanercept (Enbrel; Immunex, Thousand Oaks, CA, USA) was administrated i.v. (0.2 ml, 6 mg/ml), methotrexate (Sandoz, Denmark) was administrated i.p. at 0.05 mg/kg, and Phytol (3,7,11,15-tetramethyl-2-hexadecene-1-ol) was administrated s.c. (200 μl per rat).

### Flow cytometry

Blood was collected in heparinized tubes and titers of leukocytes determined on a Sysmex KX-21N cell counter. Duplicate samples of whole blood were stained with the following fluorochrome-conjugated monoclonal antibodies: anti-CD3 (1F4), anti-CD4 (OX35), anti-granulocytes (His48), and anti-CD8 (OX8). For analyses of IFNγ and IL-17-producing T cells after treatment with anti-IL-17-neutralizing antibodies, 10^6^ splenocytes were resuspended in 100 μl complete DMEM medium (described above) without ConA and transferred to 96-well u-bottom plates. The cells were mixed with an equal volume of the same medium containing 10 ng/mL Phorbol 12-Myristate 13-Acetate (PMA), 0.6 μg/mL ionomycin, and 10 μg/mL Brefeldin A (all from Sigma-Aldrich) and incubated for 4 h at 37 °C in a 5% CO2 atmosphere. PMA-stimulated cells were washed in cold EDTA-FACS buffer, stained with antibodies (described above) and then fixed and permeabilized in Cytofix/Cytoperm (BD Biosciences) for 30 min at RT. Cells were then stained intracellularly with antibodies against IL-17 (IC421P, purchased from R&D Systems) and IFNγ (DB-1, BD Pharming). Fluorescence-minus-one (FMO) controls were used in all panels. Dead cells were excluded from the analysis using LIVE/DEAD violet (Invitrogen, Carlsbad, CA, USA). Acquisition was made on a Becton Dickinson SORP BD LSRII Analytic Flow Cytometer and the data was analyzed with FlowJo (Tree Star Inc., Ashland, OR, USA).

### Statistics

Comparisons of incidence were evaluated by Fischer’s exact test. Clinical scores and quantitative PCR were analyzed using the Mann-Whitney *U* test or Kruskal-Wallis test with a Dunn’s post-test (for quantitative PCR analyses). All analyses were performed using Graphpad Prism software (La Jolla, CA, USA). In all experiments, a *P* value of less than 0.05 was considered significant.

## Results

### CD4^+^ T cells from lymph nodes, but not spleen, transfer chronic arthritis

**Fig. 1:**
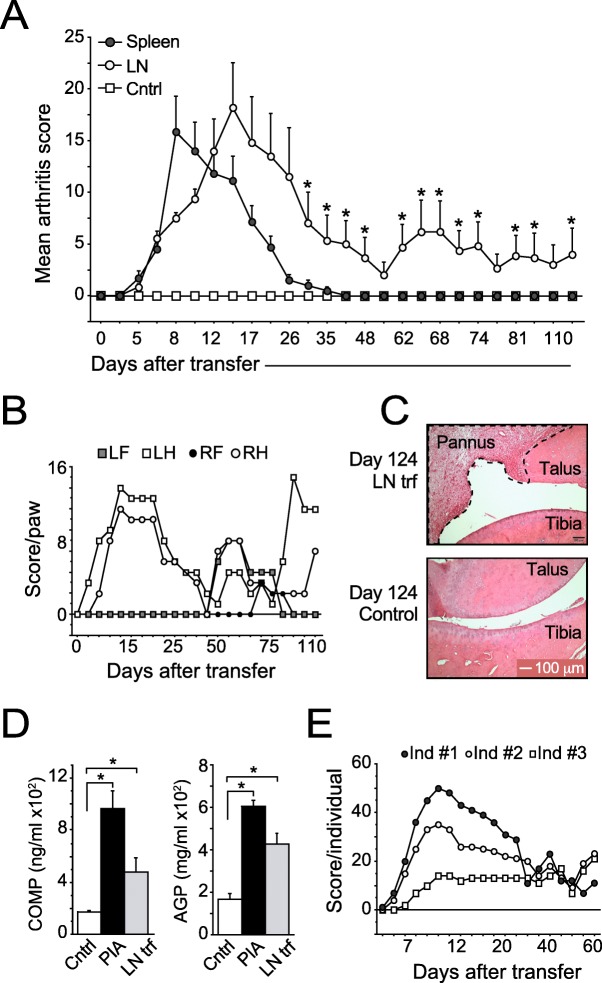
Joint-draining lymph node-derived CD4^+^ T cells from pristane-injected rats induce chronic arthritis. **a** Arthritis development in irradiated rats transferred with 2 × 10^7^ in vitro-re-stimulated cells from the LN or spleen of pristane-injected donors (14 days after pristane injection). Control rats were transferred with an equal number of non-re-stimulated LN cells. *n* = 4 rats/group. **b** Chronic relapses of arthritis in individual paws of a representative recipient transferred with re-stimulated LN cells. *n* = 1. **c** H&E staining of a representative arthritic hind paw (top) showing typical pannus formation above the joint cavity at day 124 after injection of re-stimulated cells from LNs of pristane-injected donors. Bottom image shows a corresponding section from a rat transferred with non-re-stimulated cells. **d** Serum levels of COMP and AGP on day 124 post-transfer. Control, rats transferred with non-re-stimulated LN cells; PIA, rats with chronic PIA (non-transferred). *n* = 4–6/group. **e** Arthritis development in irradiated rats transferred with 2 × 10^7^ in vitro-re-stimulated CD4^+^ T cells. *n* = 3. Data show mean values ± SD. Statistical analyses using the Mann-Whitney *U* test; *< 0.05, **< 0.01. RH, right hind paw; LH, left hind paw; RF, right front paw; LF, left front paw

In contrast to the high incidence of chronic arthritis in rats injected with pristane [17], the disease induced by the adoptive transfer of spleen-derived T cells from pristane-injected rats is acute and resolves spontaneously after 4–5 weeks [21]. Given that lymph from the hind legs preferentially enters the inguinal lymph nodes (in addition to the popliteal lymph nodes) [28], we set out to examine whether inguinal lymph node (hereafter referred to as LN)-derived T cells would be more arthritogenic than T cells derived from the spleen. Transfer of in vitro-reactivated T cells from pristane-injected donors into syngeneic, irradiated recipients revealed no difference in the arthritogenic potency between LN- and spleen-derived T cells during the first 4–5 weeks after transfer (Fig. 1a). However, following an almost complete remission, the arthritis relapsed in rats transferred with LN-derived, but not spleen-derived, T cells (Fig. 1a, b), and the histological examination at the end of the experiment (day 124) demonstrated that several, albeit not all, of the rats transferred with LN-derived T cells had joints with severe pannus formation (Fig. 1c). In addition to the clinical and histopathological manifestations, serum from rats that had received LN-derived T cells had elevated levels of cartilage oligomeric matrix protein (COMP) at day 124 post-transfer, indicating an active and ongoing cartilage degradation, as well as alpha-1-acid glycoprotein (AGP), an acute-phase protein whose levels are highly correlated with that of clinical arthritis in PIA [17, 18, 20] (Fig. 1d). Although the in vitro*-*reactivated LN cells consisted of both CD4^+^ and CD8^+^ T cells, only MHCII-restricted CD4^+^ T cells have been shown to be arthritogenic in PIA [20, 21], and, similar to bulk T cells, purified CD4^+^ T cells induced chronic arthritis when transferred into irradiated recipients (Fig. 1e).

Taken together, LN-derived, but not spleen-derived, CD4^+^ T cells were able to induce chronic relapsing arthritis upon transfer into irradiated rats. The chronic disease lasted for at least 4 months and was accompanied by elevated serum levels of cartilage and acute-phase proteins.

### Impact of host-derived T cells on acute and chronic arthritis

T cells from LNs of pristane-injected rats were able to induce chronic arthritis. However, the arthritis was notably mild compared to PIA, in particular in its chronic phase, suggesting that, over time, donor T cells lost most of their arthritogenic potency. Such gradual loss of arthritogenicity could be due to T cell exhaustion or arise from suppression imposed by de novo generation of host T cells [29, 30].

**Fig. 2:**
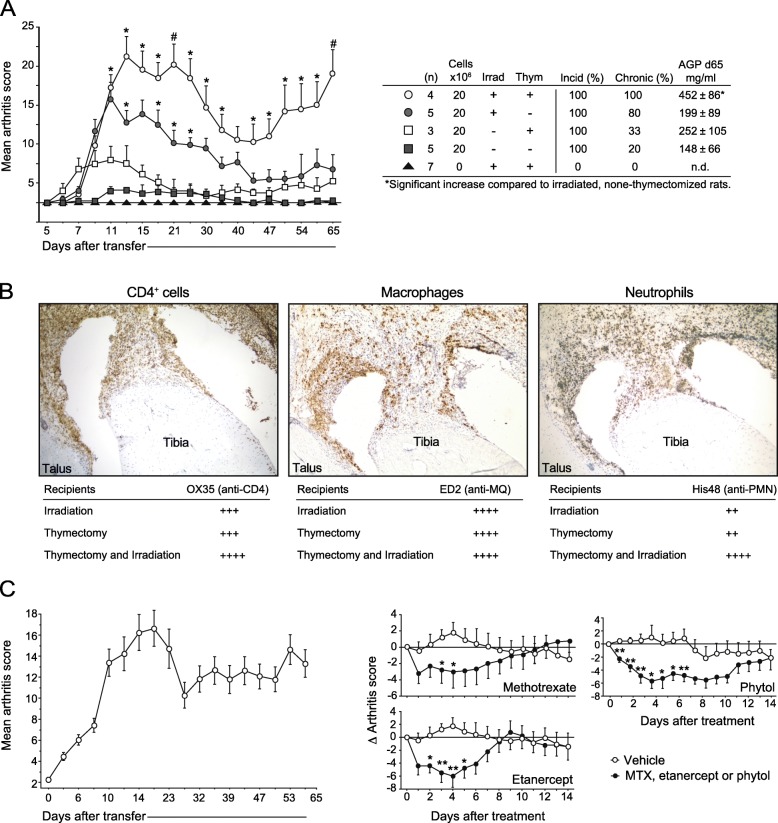
Irradiation in combination with thymectomy enhances chronic arthritis. **a** Rats were pre-conditioned by irradiation and/or thymectomy (as indicated in the enclosed table) prior to transfer. Left: arthritis development in rats transferred with or without LN-derived cells. Right: summary data of total incidence (Incid), incidence of arthritis lasting > 40 days (Chronic), and level of acute-phase protein (AGP). Number of rats per group is indicated in the enclosed table. **b** Immunohistochemistry of hind paws from rats shown in **a** stained for CD4^+^ cells, macrophages and neutrophils. Bottom tables show quantifications (as percent of total cells) of infiltrating cell populations; 0.5 to 5% (++), 5 to 20% (+++), or 20 to 50% (++++). **c** Irradiated/thymectomized rats (*n* = 70) were transferred with T cells as in **a**. On day 65 post-transfer, rats were divided into three disease-matched groups that were treated with methotrexate (*n* = 9), etanercept (*n* = 9), or phytol (*n* = 8). Filled and open circles represent normalized disease scores (vs. day 65) for treated and none-treated individuals, respectively. Statistical analyses as in Fig. 1. All groups in **a** were compared to non-irradiated, non-thymectomized rats except those indicated by a hash sign (#), which were compared to irradiated, non-thymectomized recipients. Data shown in **c** were pooled from two separate experiments

Although irradiation alone is sufficient to deplete the vast majority of circulating mature T cells (data not shown), by 40–50 days, most peripheral T cells would have returned to their normal levels [31]. To assess whether the reappearance of host-derived T cells impacted the development and progression of arthritis, we repeated the experiment but thymectomized the recipients prior to irradiation and cell transfer. Compared with only irradiation or thymectomy, the combination of irradiation/thymectomy increased the severity of acute (< 40 days after transfer) and chronic (> 40 days after transfer) arthritis (Fig. 2a*,* left panel). In addition, serum from irradiated and thymectomized rats demonstrated elevated levels of AGP compared with serum from rats only irradiated or only thymectomized (Fig. 2a, right panel). In addition, the immunohistological examination of hind paws on day 65 after transfer revealed that irradiation in combination with thymectomy increased the infiltration of CD4^+^ cells and neutrophils in the synovium, whereas the presence of macrophages was similar between the groups (Fig. 2b). Thus, the return of host-derived T cells appeared to curtail the progression of chronic arthritis in T cell-transferred rats.

PIA is a valuable model for studying how anti-inflammatory and immune-modulating drugs impact chronic inflammation [17]. The establishment of a chronic arthritis transfer model provided an opportunity to assess the potency of conventional RA therapy in an adjuvant-free environment. We therefore set out to test two standard-of-care therapies, etanercept (anti-TNF) and MTX, and a novel drug, phytol, a reactive oxygen species-inducer with immune-modulating functions, that is highly potent in PIA [32], in rats with chronic transferred arthritis. Compared with vehicle (PBS) alone, all of these drugs were able to suppress the progression of arthritis in irradiated/thymectomized rats when injected on day 65 post T cell transfer (Fig. 2c).

Together, these findings suggest that de novo-generated host-derived T cells acted to suppress the arthritogenic potency of donor T cells, thereby limiting the development of chronic arthritis. T cell exhaustion did not seem to be causing the diminished arthritogenicity. In addition, these results show that T cell transfer of chronic arthritis may provide a useful model for understanding the mechanisms of immune-modulating drugs in chronic inflammation.

### CD4^+^ T cells in draining LNs express high levels of cytokines associated with arthritis progression

**Fig. 3:**
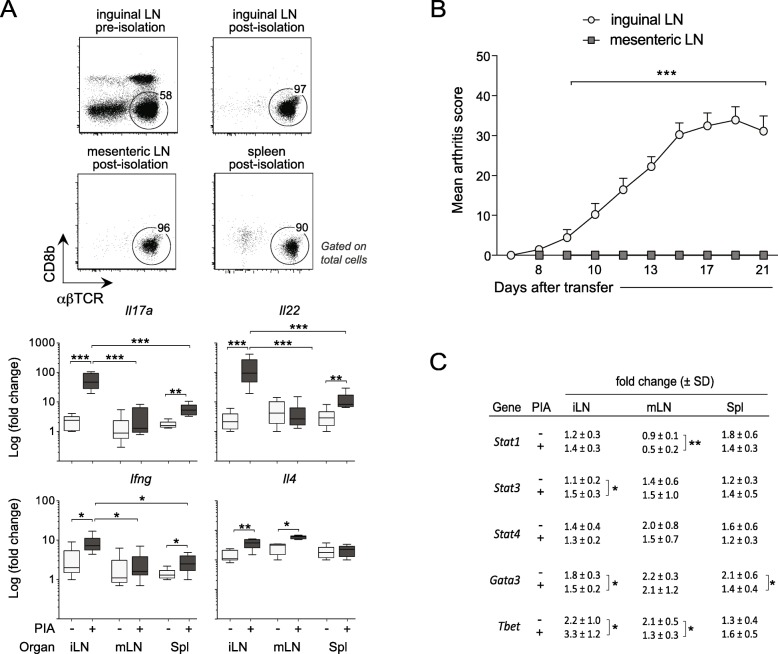
The ability of CD4^+^ T cells to transfer chronic arthritis correlates with their expression of Th17-associated cytokines. **a** Top: representative flow cytometric plots of CD4^+^ T cells from various lymphoid organs of pristane-injected rats (14 days after pristane injection) before and after in vitro isolation. Bottom: transcript levels, determined by quantitative RT-PCR and depicted as fold change (FC), of various cytokines in CD4^+^ T cells from lymph node and spleen, before (–) and 14 days after (+) injection of pristane. The expression of each target gene was normalized to the geometric mean of the expression of *Arbp*, *Gusb,* and *Actb*. *n* = 4 rats/group. **b** Arthritis development in rats transferred with 2 × 10^7^ in vitro-re-stimulated cells from inguinal or mesenteric LNs (*n* = 5–9 rats/group) of pristane-injected donors. **c** Corresponding data (as in **a**) for various transcription factors. Box and whisker plots in **a** show upper and lower quartiles (the outer boundaries of the box), median (horizontal line inside box) and highest and lowest observations (whiskers). Data in **c** shows fold change ± SD. Statistical analyses using the Mann-Whitney *U* test; *< 0.05, **< 0.01.1, ***< 0.001. iLN, inguinal lymph nodes; mLN, mesenteric lymph nodes; Spl, spleen

Based on our previously published data showing that IFN-γ-producing Th1 cells are critical for the early inflammatory response in PIA while IL-17 are more important for the perpetuation of disease [24], we set out to explore the expression of various cytokines and transcription factors in CD4^+^ T cells from LN and spleen. On day 13 after pristane injection, mRNA expression of *Il17* and the Th17-associated cytokine, *Il22,* was approximately 60–80-fold higher in CD4^+^ T cells from inguinal than mesenteric LNs (Fig. 3a), which drains the intestine but not the joints, and whose T cells do not transfer arthritis (Fig. 3b). Furthermore, the expression of *Il17* and *Il22* was higher in inguinal LN- than spleen-derived CD4^+^ T cells from pristane-injected rats, although both transcripts were more highly expressed in spleen-derived CD4^+^ T cells of pristane-injected compared with naive rats (Fig. 3a). In contrast, the expression of *Ifng* (Fig. 3a) was relatively similar between inguinal LN- and spleen-derived CD4^+^ T cells after pristane injection, and neither *Il4* (Fig. 3a) nor any of the transcription factors analyzed, *Stat1*, *Stat3*, *Stat4*, *Gata3,* or *Tbet* (Fig. 3c), showed any expressional differences that correlated with the enhanced arthritogenic potency of T cells from inguinal LNs.

In brief, CD4^+^ T cells from inguinal LNs of pristane-injected rats expressed high levels of Th17-associated transcripts, which could be important for the perpetuation of chronic PIA.

### Progression of chronic PIA is T cell- and IL-17-dependent

**Fig. 4:**
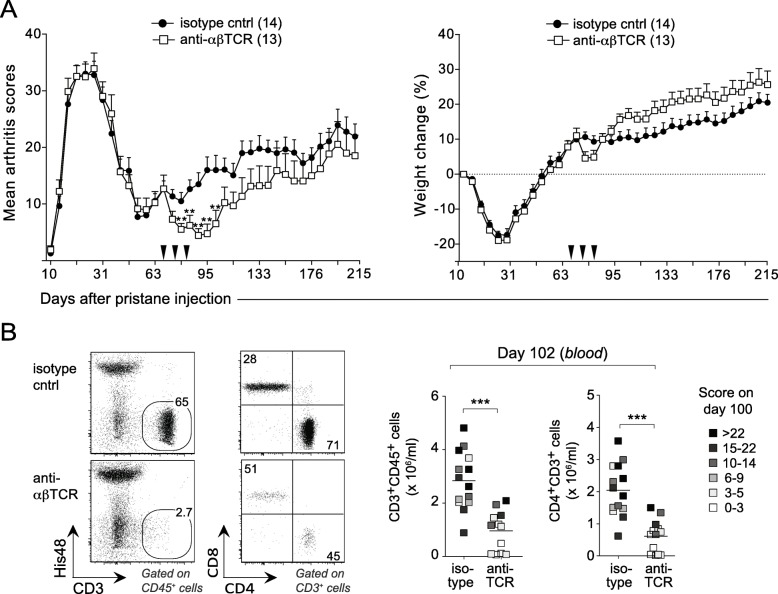
T cell ablation in rats with chronic PIA temporarily ameliorates the progression of disease. **a** Rats with early-stage chronic arthritis were treated with a mAb to αβTCR, or an isotype-matched control mAb, on day 67, 76, and 82 after pristane injection (arrow heads). Left: clinical scores. Right: relative weight change as compared to day 0. *n* = 13–14 rats/group. **b** Left: representative flow cytometric plots of peripheral blood mononuclear cells with gates depicting frequency of CD3^+^ cells (left) and CD4^+^ T cells (right) in T cell-depleted (bottom) and non-depleted (upper) rats on day 102 after pristane injection. Right: summary data of CD3^+^ and CD4^+^ T cell titers. The shading of the symbols represents the severity of arthritis on day 100 after pristane injection according to the enclosed legend. Data in **a** show mean values ± SD. Horizontal line in **b** depicts mean values. Statistical analyses as in Fig. 1

Given that CD4^+^ T cells from LNs of pristane-injected rats were able to induce and sustain arthritis in lymphopenic recipients for well over 100 days, we decided to revisit the question of whether T cells were critical for the perpetuation of chronic arthritis in PIA. A cohort of rats with early-stage chronic arthritis was treated with an anti-αβTCR mAb, or an isotype-matched control mAb, on day 67, 76, and 82 after pristane injection. Within days after the first mAb injection, anti-αβTCR mAb-treated rats went into remission, which lasted for about 3–4 weeks (Fig. 4a). Flow cytometry analyses of PBMCs on day 102 after pristane injection revealed that rats treated with anti-αβTCR mAb had significantly lower titers of total CD3^+^ leukocytes and CD4^+^ T cells compared with rats treated with an isotype-matched control mAb (Fig. 4b). Moreover, these T cell titers correlated with the severity of arthritis, suggesting that the arthritis remission was indeed a direct consequence of T cell depletion.

**Fig. 5:**
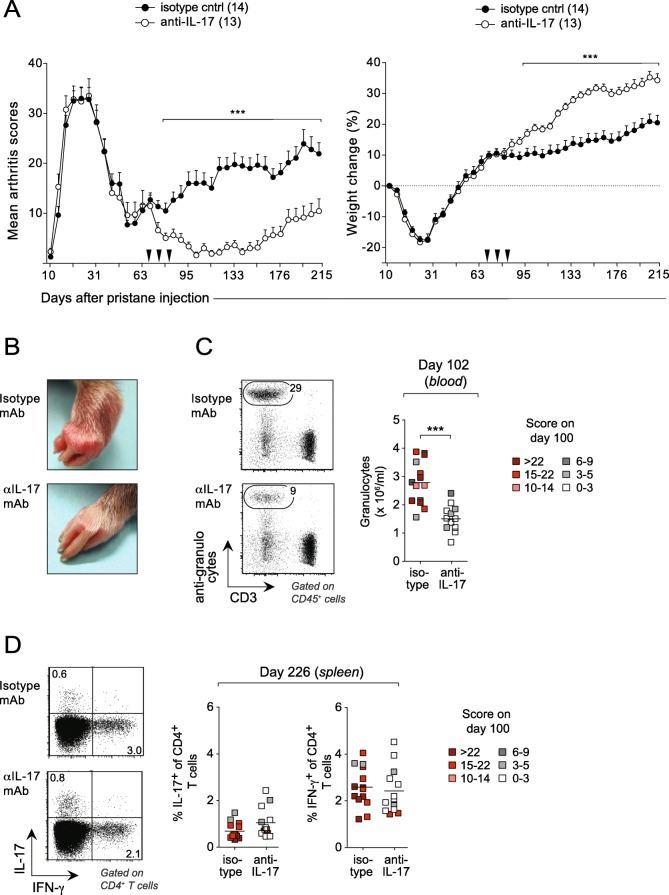
Neutralization of IL-17 efficiently blocks the progression of chronic disease in PIA. **a** Rats, which were part of the same cohort as those shown in Fig. 4, were treated with a mAb to IL-17, or an isotype-matched control mAb, on day 67, 76, and 82 after pristane injection (arrow heads). Left: clinical scores. Right: relative weight change as compared to day 0. *n* = 13–14 rats/group. **b** Representative images of front paws of rats injected with pristane (day 102 after injection) and treated with either IL-17 depleting or isotype control antibodies. **c** Left: representative flow cytometric plots of peripheral blood mononuclear cells with gates depicting frequency of granulocytes in IL-17-depleted (bottom) and non-depleted (upper) rats on day 102 after pristane injection. Right: summary data of granulocyte titers. **d** Representative flow cytometric plots and summary data for the percentage of IL-17^+^ and IFNγ^+^ CD4^+^ T cells on day 226 in the spleen. The colors of the symbols in **c** and **d** represent the severity of arthritis on day 100 after pristane injection according to the enclosed legends. Data in **a** show mean values ± SD. Horizontal line in **c** and **d** depicts mean values. Statistical analyses as in Fig. 1

T cells from inguinal LNs expressed higher levels of *Il17* than T cells from the spleen (Fig. 3a). To probe whether the production of IL-17 was associated with the development of chronic PIA, a third set of rats were treated with anti-IL-17 mAb on day 67, 76, and 82 after pristane injection. The anti-IL-17-treated rats went into remission shortly after Ab injection but, in contrast to rats treated with anti-αβTCR mAbs, remained in remission or had only a few relapses for the entire duration of the experiment (> 200 days) (Fig. 5a, b). Flow cytometry analyses on day 102 after pristane injection showed that rats treated with anti-IL-17 mAb had significantly reduced levels of neutrophils in the blood compared with controls (Fig. 5c), while there was no difference in the proportion of IL-17 or IFN-γ-producing CD4^+^ T cells in the spleen at the end of the experiment (day 226) (Fig. 5d).

Taken together, depletion of T cells resulted in a rapid, albeit transient, amelioration of chronic arthritis, suggesting that T cells are important for the perpetuation of chronic PIA. Compared with T cell depletion, the impact of neutralizing IL-17 on chronic arthritis was more potent and long-lasting.

## Discussion

The question of whether and how T cells are actively participating in the chronic inflammatory response in RA is debated. Several studies have shown that RA synovial tissue is enriched in activated T cells [33–35], but whether those T cells contribute to sustaining the inflammation is unclear. Moreover, in many rodent models of RA, T cells are required for the induction but not the perpetuation of disease [9–13]. Here, we demonstrate that transfer of T cells from joint-draining LNs of pristane-injected rats into lymphopenic recipients induces a type of arthritis that essentially phenocopies the relapsing-remitting disease course of PIA. In addition, rats with late-stage chronic PIA temporarily recovered from arthritis when treated with T cell-depleting antibodies. Taken together, these findings suggest that T cells play an important role during the chronic stage of PIA.

A number of pre-conditioning regimens were required to enhance the efficiency of the adoptive T cell transfer. First, pre-conditioning of recipient rats by gamma radiation and thymectomy was important for the induction of chronic arthritis. While lymphoablation through irradiation will allow donor T cells to expand in the host, over time, such expansion, known as lymphopenia-induced proliferation (LIP), is dampened as new T cells egress from the thymus. Regulatory T (Treg) cells are particularly important to suppress LIP [36]. Treg cells are also known to undergo extensive proliferation under semi-lymphopenic conditions as long as the levels of IL-2 are sufficient [37, 38], which may explain why they are typically overrepresented in the peripheral T cells pool after lymphodepletion [39]. Such expansion of Treg cells under lymphopenic conditions induces a state of generalized immune suppression, which may explain why T cells from pristane-injected donors failed to induce chronic disease in irradiated, non-thymectomized rats.

Second, spleen-derived T cells turned out to be surprisingly inefficient inducers of chronic arthritis. Quantitative PCR analyses revealed that CD4^+^ T cells from the spleen of pristane-injected rats expressed lower levels of Th17-associated cytokines than CD4^+^ T cells from dLNs, suggesting a difference in the proportion of Th17 cells (or in the relative expression of *Il17* and *Il22* transcripts) between the two organs. Therefore, given the importance of IL-17 in PIA [24], it is possible that spleen-, in contrast to LN-, derived T cells lack the properties necessary to sustain a chronic immune response. Moreover, as shown by Catron et al., tissue-draining LNs continue to be seeded by antigen-specific late-arriving T cells, which are enriched in central-memory cells, in the first several weeks after the primary immune response is over [40]. Such late-arriving T cells may be particularly apt in inducing chronic arthritis because of their enhanced regeneration capacity and ability to produce differentiated T cells with effector potential [41]. In this context, it is interesting to note that only T cells from joint-draining LNs were able to transfer chronic arthritis but it remains a challenge to identify the specificity and phenotype of this small subset of pathogenic cells.

The transfer experiments, discussed above, suggest that T cells contribute to the pathogenesis in chronic PIA, a notion that was further supported by T cell ablation in pristane-injected rats with chronic disease, which showed significant, albeit transient, recovery from arthritis. Although T cell depletion had limited effectiveness on disease, in particular in comparison to IL-17 neutralization, the correlation between clinical scores and T cell numbers strongly support a causative relationship between T cells and disease progression. It is worth noting that, in clinical studies, T cell-depleting strategies have generally proven to be ineffective, too, which is likely due to a combination of immunogenicity and that an extremely small number of self-reactive CD4^+^ T cells is sufficient to sustain the inflammation in RA [42]. To identify the specificity of those CD4^+^ T cells has proven to be a challenging task in humans. Here, PIA might offer a suitable surrogate system that is free from exogenous antigens that otherwise could disguise the true specificity of self-reactive T cells. As noted above, the fact that various lymphoid tissues in this model harbor CD4^+^ T cells with different potential to induce chronic arthritis might be harnessed to identify the specificity of importance for self-reactive T cells.

## Conclusions

In summary, we have demonstrated that LN-derived T cells from pristane-injected rats are able to induce chronic relapsing arthritis in lymphopenic, thymectomized recipients. The ability to transfer chronic disease correlated with a high production of Th17-associated cytokines, and neutralization of IL-17 in rats with chronic PIA efficiently dampened the progression of PIA. It is important to keep in mind that RA is not a singular disease with a defined disease etiology. Through the work in animal models, it has become clear that chronic inflammation may arise for various reasons, some involving T cells and others not, and it is likely that a similar level of heterogeneity exists in human RA.

## Supplementary information

**Additional file 1: Table S1.** Primers for expression analyses.

## Data Availability

All data and material presented are accessible upon request to the corresponding author.

## Abbreviations

AGP: a1-acid glycoprotein
COMP: Cartilage oligomeric matrix protein
i.d.: Intradermal
IL-17: Interleukin 17
LN: Lymph node
MTX: Methotrexate
PIA: Pristane-induced arthritis
RA: Rheumatoid arthritis
