# Supplementary material for: Self-reactive T cells induce and perpetuate chronic relapsing arthritis
Source: Arthritis Res Ther. 2020 Apr 28;22:95. doi: 10.1186/s13075-020-2104-7 (PMC7187533; doi:10.1186/s13075-020-2104-7)
Supplement: Supplementary file 1 — Additional file 1: Table S1. Primers for expression analyses. [file 13075_2020_2104_MOESM1_ESM.docx]

**Table S1.** Primers for expression analyses.

| *Ifng* 5’(ATTCATGAGCATCGCCAAGTTC) | *IFNg* 3’(TGACAGCTGGTGAATCACTCTGAT) |
| --- | --- |
| *IL17* 5’(CTCAGACTACCTCAACCGTTCC) | *IL17* 3’(GTGCCTCCCAGATCACAGAAG) |
| *Tbe****T*** 5’(CTGGAGCCCACTGGATGCGA) | *TBET* 3’(GACTGCAGGACGATCATCTGGGTC) |
| *Il21* 5’(GGCTGCCTGCTAAGAGGACAGG) | *IL21* 3’(CACAGGAAGGGCATTTAGCCATGTG) |
| *GATA****3* 5**’(TTGCAACGCCTGCGGACTCT) | *GATA3* 3’(TCCTCGCTGCTGACAGCCTTC) |
| *STAT3* 5’(CAGGAGGGCAGTTTGAGTCGCT) | *STAT3* 3’(CGGGGAGGTAGCACACTCCG) |
| *STAT4* 5’(CTCCCAGCCGTGCGAAGTTTC) | *STAT4* 3’(AACCCTTGTCGCCCCGTTCT) |
| *STAT1* 5’(CTCCAGGCCAAAGGAAGCACCAG) | *STAT1* 3’(ATGGGGCTGGCTTGAGGTTCTC) |
| *IL22* 5’(ATGCAGGAGGTGGTGCCTTTCC) | *IL22* 3’(TCACCGCTGATGTGACAGGGG) |
| *IL12* P40 5’(TGGGAGTACCCTGACTCCTG) | *IL12* P40 3’ (GGAACGCACCTTTCTGGTTA) |
| *GUS* 5’(CAAGGCGTCAACAAGCAT) | *GUS* 3’(CCTCCGAGTAGGGATAGTGG) |
| *Arbp* 5’(GCTTCATTGTGGGAGCAGACA) | *ARBP* 3’(CATGGTGTTCTTGCCCATCAG) |
| *Act* 5’(GGGAAATCGTGCGTGACATT) | *ACT* 3’(GCGGCAGTGGCCATCTC). |
